# Comparison of arterial and venous blood biomarker levels in chronic obstructive pulmonary disease

**DOI:** 10.12688/f1000research.2-114.v1

**Published:** 2013-04-19

**Authors:** Emer Kelly, Caroline A Owen, Amadeus Abraham, David L Knowlton, Bartolome R Celli, Victor Pinto-Plata

**Affiliations:** 1 Brigham & Women’s Hospital and Harvard Medical School, Boston, Massachusetts, 02115, USA; 2 St Elizabeth’s Medical Center, Brighton, Massachusetts, 02135, USA

## Abstract

**Purpose:** The development of novel biomarkers is an unmet need in chronic obstructive pulmonary disease (COPD). Arterial blood comes directly from the lung and venous blood drains capillary beds of the organ or tissue supplied. We hypothesized that there would be a difference in levels of the biomarkers metalloproteinase 9 (MMP-9), vascular endothelial growth factor A (VEGF-A) and interleukin 6 (IL-6) in arterial compared with venous blood.

**Methods:** Radial artery and brachial vein blood samples were taken simultaneously in each of 12 patients with COPD and seven controls with normal lung function. Circulating immunoreactive MMP-9, VEGF-A and IL-6 levels in serum were measured using quantitative enzyme-linked immunosorbent assays. Results were compared using a Student’s paired t test. The study was powered to determine whether significant differences in cytokine levels were present between paired arterial and venous blood samples.

**Results:** In the 12 patients with COPD, four were female, and age ranged 53-85 years, mean age 69 years. Three patients in the control group were female, with age range 46-84 years, mean age 64.7 years. In the COPD group, three patients had mild, five moderate and four severe COPD. No significant difference was found between arterial and venous levels of MMP-9, VEGF-A or IL-6.

**Conclusions:** In this pilot study, levels of the measured biomarkers in arterial compared with venous blood in both COPD patients and healthy controls did not differ. This suggests that as we continue to chase the elusive biomarker in COPD as a potential tool to measure disease activity, we should focus on venous blood for this purpose.

## Introduction

Chronic obstructive pulmonary disease (COPD) is a major public health issue, predicted to become the 3 ^rd^ leading cause of mortality in the United States ^1, 2^. At present, the Global Initiative for Obstructive Lung Diseases (GOLD) divides patients into categories of mild, moderate, severe and very severe based on the forced expiratory volume in 1 second (FEV _1_). This classification has been shown to predict outcome and has been pivotal in guiding treatment of the disease ^3^.

The progression of COPD has classically been determined by the change of the FEV _1_ over time, usually measured over years. Indeed, the duration of the large trials that have evaluated the effect of pharmacological or surgical benefits in patients with COPD have ranged from 2 to 4 years ^4– 7^. It follows that defining disease activity or response to therapy with validated biomarkers that reflect disease progression would make potential future interventions easier to evaluate. Several studies have reviewed the roles of different potential biomarkers in COPD ^8^. Our group has recently shown that serum levels of interleukin 6 (IL-6) and tumor necrosis factor alpha (TNF alpha) were higher and matrix metalloproteinase 9 (MMP-9) and vascular endothelial growth factor (VEGF) lower in patients with severe and very severe COPD compared with smoker and non-smoker controls without airflow obstruction ^9^. Furthermore, the levels correlated with several phenotypic expressions of the disease including exercise capacity, quality of life, exacerbation and mortality ^9^. Data from the ECLIPSE investigators, who studied a larger cohort of COPD patients, showed that several biomarkers correlated with baseline FEV _1_ but only one correlated with rate of decline in FEV _1_, the Clara-Cell Protein 16 (CC-16) ^10^. While there is no question that a biomarker would be of great importance in COPD, the sample site having the greatest yield has not been clearly defined ^11^.

Blood from the systemic circulation returns to the lung for gas exchange and, theoretically, the presence of biomarkers could be modified during the transient time blood spends in the pulmonary vessels. Arterial blood should provide a more direct window to events occurring in the lungs than venous blood, which is perhaps more reflective of events happening in the capillary bed of the organ/tissues that it supplies. This is most evident in the significant differences that exist between arterial and venous blood gas measurements.

We conducted this pilot study to test the hypothesis that in patients with COPD (a primary disease of the lungs) there is a difference in serum concentrations of the biomarkers MMP-9, VEGF-A and IL-6 between simultaneously collected arterial and venous blood. Further, we compared the results with similar samples from patients without airflow obstruction that served as controls.

## Materials and methods

### Study population

This was a prospective pilot study. Samples were collected from patients attending the pulmonary clinic at St. Elizabeth’s Medical Center in Boston, Massachusetts. The study was approved by the Human Institutional Review Board of the institution and all patients signed the informed consent. The patients in the COPD group had smoking history ≥ 20 pack-years (1 pack year is equivalent to 1 year at 20 cigarettes per day) and had post-bronchodilator FEV _1_/FVC (forced vital capacity) ratio, < 0.7 after 400 μg of inhaled salbutamol. Patients had stable COPD and were not included if they had a history of an exacerbation in the last 3 months. The controls were patients attending the pulmonary clinic at the same institution with no history of COPD and normal lung function.

### Clinical parameters

Age, gender, smoking history and body mass index (BMI) were recorded for every participant. All subjects performed a spirometry according to the American Thoracic Society (ATS) recommendations and standard references and severity of COPD was categorized using GOLD staging. The BODE score is a composite score of BMI, degree of obstruction as recorded by FEV _1_, dyspnoea as quantified by the modified Medical Research Council dyspnoea scale and exercise tolerance, as measured with a 6 minute walk test ^12^.

### Biomarker analysis

Simultaneous radial artery and antecubital venous samples were obtained in each participant. The blood samples were centrifuged immediately at 2500 rpm for 10 minutes and serum stored at -80ºC. Circulating immunoreactive MMP-9, VEGF-A and IL-6 levels in the serum were measured using commercially available quantitative enzyme-linked immunosorbent assays (ELISA) (Human IL-6 ELISA Ready-Set-Go! eBioscience, San Diego, CA; Human total MMP-9 DuoSet, R&D Systems, Inc. Minneapolis, MN; Human VEGF-A Platinum ELISA from eBioscience, San Diego, CA).

### Statistical analysis

We worked out a power/sample size calculation based on the premise that this was a study of a continuous response variable from matched pairs of study subjects. Prior data indicated that the difference in the response of matched pairs is normally distributed with standard deviation of 0.3 in the case of MMP-9 and VEGF-A, and 0.4 for IL-6 ^13^. If the true difference in the mean response of matched pairs is 0.3, we would need to study 10 pairs of subjects to reject the null hypothesis with probability (power) of 0.8. If the true difference in the mean response of matched pairs is 0.4, we would need to study 12 pairs of subjects to be able to reject the null hypothesis with a probability (power) of 0.8. The Type I error probability associated with this test is 0.05.

Groups were compared using the Student’s t test for normally distributed variables and Mann-Whitney U test for variables not normally distributed. p ≤ 0.05 was considered statistically significant. Spearman’s rank order correlation coefficient was also used for non-parametric data. Statistical analysis was performed using a commercial statistical package (Sigma Stat, Sigma Plot).

## Results

The characteristics of all participants are shown in Table 1. The twelve patients in the COPD group (4 female) ranged in age between 53 and 85 years. Three had mild, five had moderate and four had severe COPD by the GOLD classification ^14^. The seven patients (three females) included in the control group had normal lung function and their mean age range was similar to the COPD group (46–84 years). The majority of subjects in both groups were ex-smokers, with only 3 patients in the control group being never-smokers.

**Table 1.** Patient demographics.

Normal	Age (years)	Gender (Male-M, female-F)	Smoking history (Smoker-S, Non-smoker-NS, Ex-smoker-ExS)	FEV _1_ (liters)	FEV _1_ (% predicted)	FVC	FEV _1_/FVC	Stage of COPD	BMI	6 minute walk test (meters)	BODE score
**1**	46	M	NS	4.79	128%	5.72	84	Normal	23	495	0
**2**	46	M	NS	4.35	95%	5.88	74	Normal	28	690	0
**3**	66	F	NS	1.79	83%	2.29	78	Normal	28.3	425	0
**4**	73	F	ExS	2.33	109%	3.02	77	Normal	39	330	2
**5**	80	F	ExS	2.16	95%	3.03	71	Normal	37	370	2
**6**	58	M	ExS	2.59	87%	4.08	70	Normal	–	–	–
**7**	84	M	ExS	1.5	73%	2.13	70	Normal	–	Refused	–
COPD											
**1**	80	M	ExS	2.24	85%	3.66	61	Mild	26.5	380	0
**2**	65	F	ExS	2.23	87%	3.68	61	Mild	29	423	0
**3**	71	F	ExS	2.11	104%	3.45	61	Mild	26.6	450	0
**4**	53	M	S	2.33	68%	3.87	60	Moderate	27	505	0
**5**	68	M	ExS	2.06	62%	4.43	46	Moderate	27.5	535	1
**6**	76	M	ExS	1.71	60%	3.43	50	Moderate	34	403	1
**7**	85	M	ExS	1.68	67%	2.42	68	Moderate	26.9	287	2
**8**	78	M	ExS	1.6	62%	3.47	46	Moderate	27	460	1
**9**	66	F	ExS	0.97	40%	2.47	40	Severe	28	505	2
**10**	65	M	S	1.45	39%	4.69	31	Severe	29	377	2
**11**	57	M	ExS	1.41	42%	3.36	42	Severe	33.9	431	3
**12**	64	F	ExS	2.34	36%	2.78	63	Severe	33	362	2

### Arterial and venous biomarker levels

**Figure 1.**
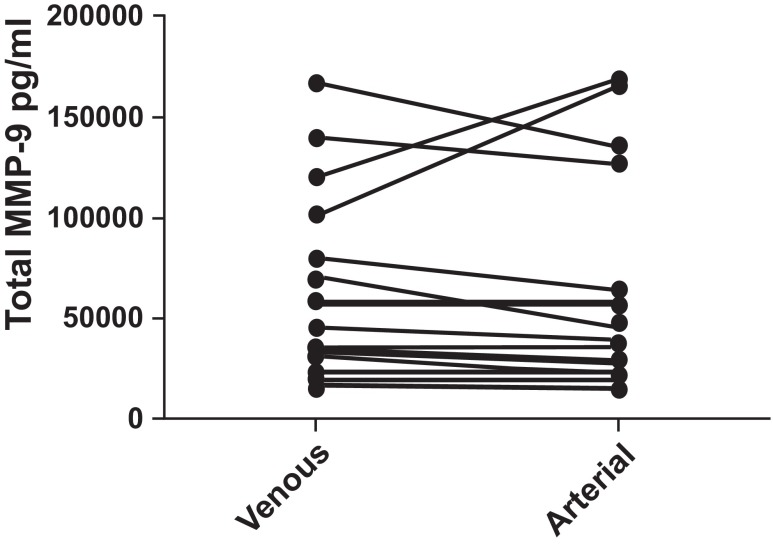
Total human MMP-9 levels were compared between paired samples of venous and arterial blood from COPD patients and normal controls. No difference was found (p = 0.812). Pairing of the two groups was shown to be effective with rs (Spearman, Approximation) 0.8782 and p<0.0001.

**MMP-9 ( Figure 1):** Serum MMP-9 levels were not normally distributed in all of the subjects (n =19). The median venous MMP-9 level was 40,706 pg/ml, (interquartile range 23,659–79,595 pg/ml). The median arterial level was 37,653 pg/ml (interquartile range 21,833–64,351 pg/ml). There was no significant difference between the venous and arterial levels, p = 0.812. There was no difference between COPD and control levels of venous or arterial MMP-9.

**Figure 2.**
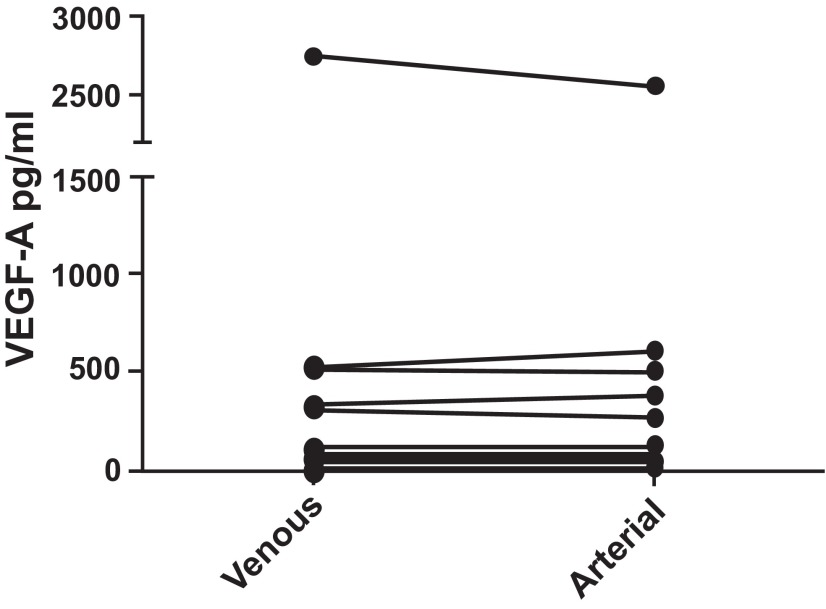
VEGF-A levels were compared between paired samples of venous and arterial blood from COPD patients and normal controls. No difference was found (p = 0.550). Pairing of the two groups was shown to be effective with rs (Spearman, Approximation) 0.7526 and p = 0.0001.

**VEGF-A ( Figure 2):** In the 19 subjects, the VEGF-A levels in venous blood had a median value of 67.24 pg/ml, with interquartile range 45.24 to 268.90 pg/ml whereas in arterial blood the median value was 89.88 pg/ml, with interquartile range 58.90 to 239.38 pg/ml. There was no statistically significant difference between arterial and venous samples (p = 0.249). No significant difference was observed between patients with COPD and controls.

**Figure 3.**
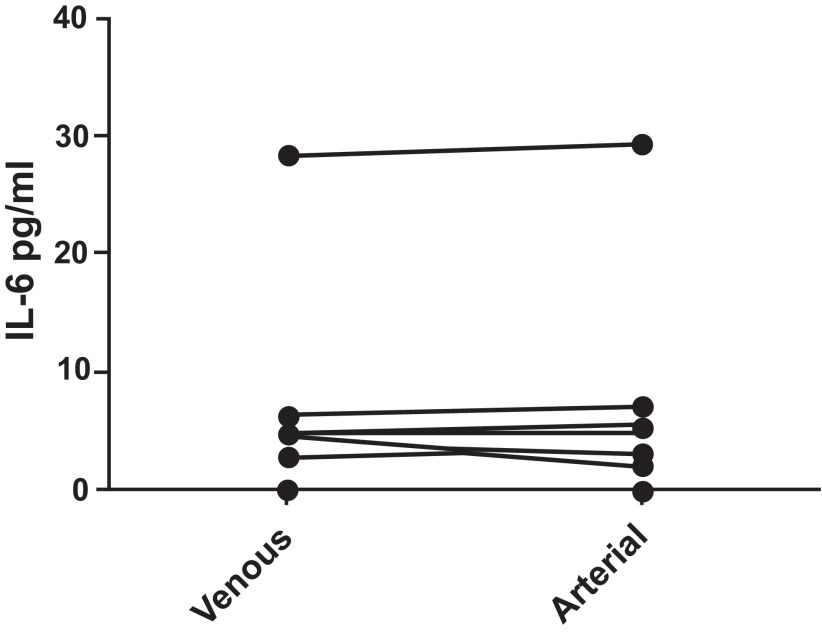
IL-6 levels were compared between paired samples of venous and arterial blood from COPD patients and normal controls. No difference was found (p = 0.986). Pairing of the two groups was shown to be effective with rs (Spearman, Approximation) 0.9966 and p<0.0001.

**IL-6 ( Figure 3):** IL-6 levels were undetectable in many of the subjects. The median venous level of IL-6 was 0, with an interquartile range of 0 to 4.52 pg/ml and the median arterial level was also 0 pg/ml with an interquartile range of 0 to 3.61 pg/ml. There was no statistically significant difference (p = 0.986) between venous and arterial levels of this cytokine. As levels of IL-6 were undetectable in many of the patients, it was not possible to determine if a significant difference existed between those with COPD and the control group.

## Discussion

This pilot study showed no difference in serum levels of MMP-9, VEGF-A or IL-6 between arterial and venous blood samples in a group of patients with COPD and controls without airflow obstruction. Our results indicate no advantage of obtaining arterial over venous samples for the determination of these biomarker levels in patients with COPD.

At present, the most important determinant of COPD severity and progression is the FEV _1_ value and its progression over time, usually measured in years. This approach has its limitations and fails to account for the so-called activity of the disease ^15^. As more treatments for COPD are emerging, the importance of biomarkers that reflect disease activity becomes paramount. A biomarker, by definition, is “any molecule or material (e.g. cells and tissues) that reflects the disease process” ^16^. Because of its ease of accessibility, work has focused on peripheral blood biomarkers. Many have been investigated with initial work showing C reactive protein (CRP) levels to be associated with degree of airway obstruction, although subsequent work in patients with moderate to severe COPD found no relationship between CRP levels and reduced survival ^17– 19^. Other promising biomarker candidates previously studied include circulating levels of Clara cell secretory protein-16 (CC-16) ^20^, surfactant protein (SP)-D ^21^ and serum amyloid A (SAA) ^22^. Serum levels of CC-16, a marker of Clara cell toxicity, are reduced in patients with COPD ^10^, while SP-D is increased in smokers with and without COPD ^21^, and SAA may be a potential biomarker of COPD exacerbation ^22^.

There are no prior published results on cytokine measurement in arterial blood in COPD patients. Studies where arterial vessels are accessed for cardiopulmonary bypass or for extracorporeal liver support have measured arterial blood levels of various cytokines but only within narrow inclusion criteria, and arterial and venous level comparisons were not published ^23, 24^. In a study of the effect of moderate hypothermia on systemic and internal jugular plasma IL-6 levels after traumatic brain injury in humans, IL-6 was found to be significantly higher in internal jugular venous blood than in arterial plasma ^25^. In the setting of recent neuro-trauma this is understandable. Having stressed the importance of suitable potential biomarkers in COPD, we hypothesized that determining the optimal source of the sample could be of potential value. Our most important finding was that for the biomarkers studied, levels did not differ between arterial and venous samples. This was not due to the nature of the biomarkers selected because the markers we investigated in this study were chosen based on prior work demonstrating the level of IL-6 was higher and MMP-9 and VEGF levels lower in patients with more advanced COPD compared with controls ^9, 26, 27^. Although the host response to insult is, to a large extent, compartmentalized to the affected lung, cytokine spillover into the systemic circulation has been shown to occur ^10, 28^ and be measurable in the systemic circulation ^10, 28^. We did not find differences in serum biomarker levels between patients with COPD and those without airflow obstruction; however, the study was not powered to explore this hypothesis.

Our pilot study has some limitations. First, the number of patients recruited could be considered small, but it was powered to address whether there was a difference between arterial and venous levels of MMP-9, VEGF-A and IL-6. The use of matched samples allowed for accurate interpretation of the results with these subject numbers. Second, the blood samples were drawn from the radial artery and from a peripheral antecubital vein. Possibly, to obtain more accurate sampling of blood immediately leaving the lung, pulmonary arterial sampling would have been optimal. However, sampling of central venous blood is invasive and would not offer any practical advantage. Third, it is possible that the 3 selected analytes are not "exclusively" produced in the lung (as it is the case for SPD and CC16) and represent the systemic compartment and not just the lung milieu. This possibility requires the simultaneous measurement of these biomarkers in future studies.

In summary, this pilot study shows that there was no difference in levels of MMP-9, VEGF-A or IL-6 when measured in blood samples from the radial artery compared with peripheral venous samples. This suggests that as we continue to chase the optimal biomarker in COPD as a potential tool to measure disease activity, focusing on venous blood for this purpose remains a valid option.
