# Supplementary material for: Comparison of arterial and venous blood biomarker levels in chronic obstructive pulmonary disease
Source: F1000Res. 2013 Apr 19;2:114. [Version 1] doi: 10.12688/f1000research.2-114.v1 (PMC3894801; doi:10.12688/f1000research.2-114.v1)
Supplement: Peer review history

# Peer review: Referee response for version 1

**Published:** 2013-06-07

**Authors:** Hajime Takizawa

**Affiliations:** 1 Department of Respiratory Medicine, University of Tokyo Graduate School of Medicine, Kawasaki City, Japan

In this pilot study, the authors compared 3 potential biomarkers between arterial and venous blood samples among normal and COPD patients. Although the methods as well as statistical analyses are sound, there exist some limitations, as discussed by the authors; such as, small number of samples, unclarified differences between pulmonary arterial and venous blood, and limited number of biomarkers. 

I believe this study becomes a milestone for the future larger-scaled study for these important topics.

I have read this submission. I believe that I have an appropriate level of expertise to confirm that it is of an acceptable scientific standard.

# Response

**Published:** 2013-09-04

**Authors:** Emer Kelly

**Affiliations:** St Vincents University Hospital, Ireland

Thank you for your comments.

# Peer review: Referee response for version 1

**Published:** 2013-05-08

**Authors:** Ravi Kalhan

**Affiliations:** 1 Asthma-COPD Program, Northwestern University Feinberg School of Medicine, Chicago, IL, USA

In this pilot study the authors evaluate whether there are differences in arterial versus venous blood in 3 potential biomarkers of COPD. Since the bulk of prior work on COPD biomarkers has been focused on venous blood and there are potential limitations to not using arterial blood. This is an appropriate study question.

The methods are appropriate, statistical analyses are sound. There is the limitation of sampling pulmonary arterial and venous blood to understand the “direct” lung effects on blood markers, but this is likely neither feasible nor practical in biomarker discovery as its clinical use would be limited.

I have read this submission. I believe that I have an appropriate level of expertise to confirm that it is of an acceptable scientific standard.

# Peer review: Referee response for version 1

**Published:** 2013-04-25

**Authors:** Rob Stockley

**Affiliations:** 1 Department of Medicine, Queen Elizabeth Hospital, Birmingham, UK

Although the article is generally well written and the methods and results are likely to be correct, there is one major flaw that really should have been addressed before embarking on this negative project; Trans lung changes in mediators means you absolutely have to obtain pulmonary arterial and peripheral arterial blood. Even then differences could reflect a “cleaning” of the blood which is a non respiratory function of the lung and is most notable in the clotting differences. 

Peripheral arterial and venous blood will if anything reflect changes in the local limb capillary circulation and clearly misses changes to more systemic venous blood that is fully mixed in the pulmonary artery. This concept was recognised over 30 years ago and although the Authors mention it in discussion as a potential limitation I think this is a definite limitation. The study also fails to include the likelihood of some shunt in COPD lungs. Finally it is not clear whether the blood was allowed to clot but as serum is mentioned the assumptionis that  it was and this would have the effect of activating local cells during the clotting process so ideally plasma should be studied.

I have read this submission. I believe that I have an appropriate level of expertise to confirm that it is of an acceptable scientific standard, however I have significant reservations, as outlined above.

# Response

**Published:** 2013-09-04

**Authors:** Emer Kelly

**Affiliations:** St Vincents University Hospital, Ireland

Thank you for taking the time to review our work. We acknowledge the limitations of this work and agree that there would be advantages to taking pulmonary arterial samples to give more physiological data. We share your concerns that the peripheral arterial sample may better reflect the occurrences in the distal capillary bed and we have raised these issues in the discussion. We were endeavouring to investigate the site of biomarker testing that could be used in real-life clinical scenarios and therefore chose radial arterial sampling. Serum was the tested sample in this study. Future work could give answers for these remaining physiological questions.
